# Dynamics of training and acute exercise-induced shifts in muscular glucose transporter (GLUT) 4, 8, and 12 expression in locomotion versus posture muscles in healthy horses

**DOI:** 10.3389/fphys.2023.1256217

**Published:** 2023-08-16

**Authors:** Carmen Vidal Moreno de Vega, Diete Lemmens, Constance de Meeûs d’Argenteuil, Berit Boshuizen, Lorie de Maré, Luc Leybaert, Klara Goethals, Jean Eduardo de Oliveira, Guilherme Hosotani, Dieter Deforce, Filip Van Nieuwerburgh, Lindsey Devisscher, Cathérine Delesalle

**Affiliations:** ^1^ Department of Translational Physiology, Infectiology and Public Health, Research Group of Comparative Physiology, Faculty of Veterinary Medicine, Ghent University, Merelbeke, Belgium; ^2^ Wolvega Equine Hospital, Oldeholtpade, Netherlands; ^3^ Department of Basic and Applied Medical Sciences, Faculty of Medicine and Health Sciences, Ghent University, Ghent, Belgium; ^4^ Biometrics Research Center, Ghent University, Ghent, Belgium; ^5^ Cargill R&D Centre Europe, Vilvoorde, Belgium; ^6^ Laboratory of Pharmaceutical Biotechnology, Faculty of Pharmaceutical Sciences, Ghent University, Ghent, Belgium; ^7^ Gut-Liver Immunopharmacology Unit, Department of Basic and Applied Medical Sciences, Liver Research Center Ghent, Ghent University, Ghent, Belgium

**Keywords:** equine, exercise, training, metabolism, glucose, glycogen, energy, fuel

## Abstract

Important changes in glucose transporter (GLUT) expression should be expected if the glucose influx plays a pivotal role in fuelling or connecting metabolic pathways that are upregulated in response to exercise. The aim was to assess GLUT4, 8, and 12 dynamics in response to training and acute exercise.

**Methods:** Sixteen untrained Standardbred mares (3-4 year) performed an incremental SET at the start and end of 8 weeks harness training. M. pectoralis (PM) and M. vastus lateralis (VL) muscle biopsies were taken before and after each SET, allowing for comparing rest and acute samples in untrained (UT) and trained (T) condition using Western Blot for GLUT quantification and Image Pro v.10 for Blot analysis. Data were normalized against GAPDH. Basal GLUT-levels of PM *versus* VL were analysed with the Wilcoxon matched-pairs signed rank test. The effect of acute exercise or training was assessed using the Friedman test with a *post hoc* Dunn’s.

**Results:** Basal GLUT4 and GLUT12 protein expression were significantly higher in the VL compared to the PM (P_GLUT4_ = 0.031 and P_GLUT12_ = 0.002). Training had no effect on basal GLUT4 expression, neither in the VL (*p* > 0.9999), nor the PM (*p* > 0.9999). However, acute exercise in trained condition significantly decreased GLUT4 expression in the VL (*p* = 0.0148). Neither training nor acute exercise significantly changed total GLUT8 protein expression. Training significantly decreased total GLUT12 protein expression in rest biopsies, only visible in the VL (*p* = 0.0359). This decrease was even more prominent in the VL after acute exercise in trained condition (P_VL_ = 0.0025).

**Conclusion:** The important changes seen in GLUT12 expression downregulation, both in response to training and acute exercise in the horse, the downregulation of GLUT4 expression after acute exercise in trained condition and the lack of differential shifts in GLUT8 expression in any of the studied conditions, questions the importance of glucose as substrate to fuel training and exercise in healthy horses. These findings encourage to further explore alternative fuels for their involvement in equine muscular energetics.

## Introduction

When looking at human or equine athletes, the same physiological paradigms apply. As a matter of fact, in athletic animals, following physical exercise, specific changes in metabolic reactions occur leading to several changes in the body, mainly in the circulatory, respiratory, endocrine, and neuromuscular systems. Changes taking place simultaneously in these systems and in an integrated manner are aimed at maintaining homeostasis in the body (Arfuso et al., 2021b; Arfuso et al., 2021a; Arfuso et al., 2021c). On top of that, in the last decade, the social interest in the welfare of the participant horses has grown substantially since the animals must face potentially stressful situations during the acts. Hence, evaluating their stress response could be the primary approach to success in the horses’ performance and wellbeing, as described in common equestrian disciplines (Bazzano et al., 2016; Arfuso et al., 2021a; Arfuso et al., 2021b; Arfuso et al., 2022a). In order to enable an athlete to achieve top performance, a whole range of conditions must be met, ranging from preferably competing in the sports disciplines for which that specific athlete harbors the most genetically favorable blueprint, to the intake of the most suitable diet to fuel the intended type of exercise, application of the optimal training protocols, as well as developing and refining a set of discipline-specific skills, such as, for example, balance, racing, or jumping capacity (Ricard and Chanu, 2001; Singer et al., 2008; Bazzano et al., 2016). With that respect, the muscular compartment, with its vast volume, plays a pivotal role. On average, it accounts for approximately 40% of the total human body mass, and in horses, depending on the breed, this proportion can be as high as 57% (Kearns et al., 2002; Klein, 2020). Due to its magnitude and involvement in physical activity, the muscular compartment governs the most important part of the body’s metabolic activity (Kaneko et al., 2008). Understanding how various fuel types are processed in this massive compartment and how they enter the myocytes and become immediately available for subsequent ATP production is essential for developing customized exercise and dietary protocols, harnessing animal welfare (Gordon et al., 2009; Fejsáková et al., 2013). After all, it is well known that most sports injuries in horses occur when horses need to engage into competition without having developed the needed performance capacity and necessary skills, leading to early onset of muscle fatigue and subsequent lack of both powerful body support and highly coordinated locomotory output, a situation that obviously catalyzes the occurrence of all kinds of different injuries (Singer et al., 2008). In addition, the muscular compartment is highly malleable, continuously adapting itself to specific types of training, leading to a different set of energy cycles that take the lead during different phases of labor. Moreover, a certain training protocol will affect a specific core set of muscles, which fulfills at that point, either a predominant posture-supporting role or a predominant locomotory role. Therefore, involving multiple muscle groups, with different physiological roles, will enhance knowledge gained by performing training studies (de Meeûs d’Argenteuil et al., 2021a; de Meeûs d’Argenteuil et al., 2021b). Likewise, exercise also has an influence on other physiological parameters such as neurohumoral factors, for example, dopamine and prolactin, having their impact on onset of fatigue, not only in response to physical effort, but also having their impact on emotional and mental stress experience levels (Arfuso et al., 2021a). Also the stress hormone cortisol, which is known to be a fuel mobilizing hormone, has been shown to increase together with the inflammation-related receptor interleukin 1 receptor antagonist in response to exercise (Arfuso et al., 2022b). Similarly, the acute phase response protein serum amyloid A (SAA) is positively correlated with the level of exercise intensity in horses (Arfuso et al., 2020). L-carnitine, an important metabolite involved in the carnitine shuttle of fatty acids into the mitochondria, has been shown to decrease immediately post-exercise and then to rise again 30 min after exercise (Arfuso et al., 2021b). Other parameters have been studied more extensively. For example the evolution of heart rate and blood lactic acid levels (Bazzano et al., 2016; De Mare et al., 2022).

There are three major fuel sources, some of which can be metabolized both aerobically and anaerobically, such as glycogen (carbohydrates; CHOs), known as the most versatile fuel type (Zierler, 1999; Klein, 2020). The two other fuels, namely lipids and proteins, can only be processed in the presence of oxygen. Because of their versatility, the current study focuses on CHOs and how they are transported into the myocytes of horses. This research group has already questioned in previous publications the importance of glycogen as pivotal fuel in horses (de Meeûs d’Argenteuil et al., 2021a; de Meeûs d’Argenteuil et al., 2021b), since, unlike in humans and other mammals, it takes a horse two to three times longer (on average 48–72 h) to replenish depleted muscular glycogen stores after extended exercise, a feature that does not match with the role of a pivotal fuel (Reed et al., 1989; Lacombe et al., 2003; Lacombe et al., 2004; Waller and Lindinger, 2010). This implies that one needs to be very careful when extrapolating one on one results from human athletic studies to horses. Moreover, in general, horses occupy a specific position regarding glucose metabolism. They are herbivores but not ruminants and are known as hindgut fermenters (Dicks et al., 2014). Hence, there is need for a better understanding of how and when glucose is transported into the equine myocytes in response to exercise and training.

Since glucose is hydrophilic, it needs specific transporter molecules to be shuttled across the lipid bilayer of the muscular cell membrane. These glucose transporters are well known as GLUTs and are a family of glycoproteins folded into 12 transmembrane α-helices with a cytoplasmatic amino (N-) and carboxyl (C-) terminus (Manolescu et al., 2007). Currently, 14 different GLUTs have been identified in humans and rodents, which are expressed in a tissue-specific manner (Manolescu et al., 2007; Thorens and Mueckler, 2010; Holman, 2020). Based on their amino acid sequence and structural similarities, the GLUT family can be subdivided into three subclasses (class I-III). Class I encompasses GLUT1, GLUT2, GLUT3, GLUT4, and GLUT14. Isotypes GLUT5, GLUT7, GLUT9, and GLUT11 are grouped into class II. Class III includes GLUT6, GLUT8, GLUT10, GLUT12, and GLUT13, which are the most recently discovered isotypes and, therefore, less well-known (Manolescu et al., 2007).

Important to notice is that not all isotypes harbor the same features; they represent differences in their 1) sensitivity to insulin/and other factors for their degree of plasma membrane expression; 2) affinity to glucose (represented as the Michaelis constant, K_m_); 3) tissue distribution; 4) cross-membranal transportation of small molecules, other than glucose; and 5) tissue expression differences between species (Mueckler and Thorens, 2013; Lacombe, 2014; Shao and Tian, 2015; Holman, 2020). Table 1 provides an overview of all the aforementioned features.

**TABLE 1:** Overview of the facilitative glucose transporter family with corresponding specific features.

Insulin-(in)dependent degree of plasma membrane expression	GLUT isotype (class)	Predominant substrate (s)	Affinity to glucose (K_m_; mM)	Tissue distribution	Other factors regulating degree of plasma membrane expression	Major species identified
Insulin-dependent	GLUT4 (II)	glucose, glucosamine	Medium (2.5–5)	Skeletal muscle, adipose tissue, cardiac muscle	Growth hormone (rat ventricular myocytes), leptin (human neuroblastoma cells), epinephrine (perfused rat heart), calcium (mouse ventricular myocytes), exercise (skeletal muscles)	Rodents, humans, horses, cows, dogs, pigs
	GLUT8 (III)	Glucose, fructose, galactose	High (∼2)	Testis, brain, blastocyst, skeletal muscle, adipose tissue, liver, spleen, lung	Adiponectin (mouse blastocysts), calcium (mouse ventricular myocytes), exercise (mouse brain and liver)	Rodents, humans, cows, horses
	GLUT12 (III)	Glucose, fructose, galactose, α-methyl-D-glucopyranoside, 2-deoxy-D-glucose	Medium (∼6.5)	Skeletal muscle, adipose tissue, prostate, cardiac muscle, placenta	TNF-α (human Caco-2 cells), exercise (human skeletal muscle)	Rodents, humans, cows, horses
Insulin-independent	GLUT1 (I)	Glucose, galactose, mannose, glucosamine	High (∼1)	Many cell types (e.g., erythrocytes, brain), blood-tissue barrier, fetal tissues	n/a^a^	Rodents, humans, horses, cows, dogs, pigs
	GLUT2 (I)	Glucose, galactose, fructose, mannose, glucosamine	Low (15–20)	Liver, small intestine, kidney, islet of Langerhans, brain	n/a^a^	Rodents, humans, horses, cows
	GLUT3 (I)	Glucose, galactose, mannose, xylose	High (<1)	Brain (neurons), testis	n/a^a^	Rodents, humans, cows, dogs, pigs
	GLUT5 (II)	Fructose	—	Small intestine, kidney, adipose tissue, brain, testis	n/a^a^	Rodents, humans, horses, cows, dogs, pigs
	GLUT6 (III)	Glucose	Low (n.d.)	Spleen, leukocytes, brain	n/a^a^	Rodents, humans
	GLUT9 (II)	Glucose, fructose, urate	High (<1)	Liver, kidney, small intestine, placenta, leukocytes	n/a^a^	Rodents, humans, dogs
	GLUT10 (III)	Glucose, galactose	High (<1)	Liver, kidney, skeletal muscle, cardiac muscle, lung, brain, pancreas, placenta	n/a^a^	Rodents, humans
	GLUT11 (II)	Glucose, fructose	Low (n.d.)	Skeletal muscle, cardiac muscle, adipose tissue, pancreas	n/a^a^	Rodents, humans
Not determined	GLUT7 (II)	Glucose, fructose	<1	Liver, small intestine, colon, prostate, testis	n/a^a^	Rodents, humans
	GLUT13 – HMIT (III)	H^+^/myo-inositol cotransporter	—	Brain, adipose tissue	n/a^a^	Rodents, humans
	GLUT14 (I) n.d./Testis n/a^a^Rodents, humans					

^a^ Knowledge is scarce; n.d. not determined; n/a not applicable.

First, not all GLUT isotypes are sensitive to insulin for their degree of plasma membrane expression. Insulin is mainly responsible for controlling glycemia by promoting the insertion of intracellular GLUTcontaining vesicles into the cell membrane via exocytosis, a process that is better known as translocation. In this regard, only plasma membrane expression of GLUT4, GLUT8, and GLUT12 is known to be governed by insulin (Shao and Tian, 2015). For the other GLUT isotypes, also other factors, such as other hormones and also ions, can control the translocation (Rattigan et al., 1991; Benomar et al., 2006; Martinez et al., 2014; Sertié et al., 2014; Gil-Iturbe et al., 2019; Burkuš et al., 2020). There are important species differences with respect to GLUT expression. All aforementioned 14 GLUT isotypes are identified in humans and rodents. However, in horses, GLUT3, GLUT6, GLUT7, GLUT9, GLUT10, and GLUT11, GLUT13, and GLUT14 are not discovered yet (Lacombe, 2014).

**FIGURE 1:**
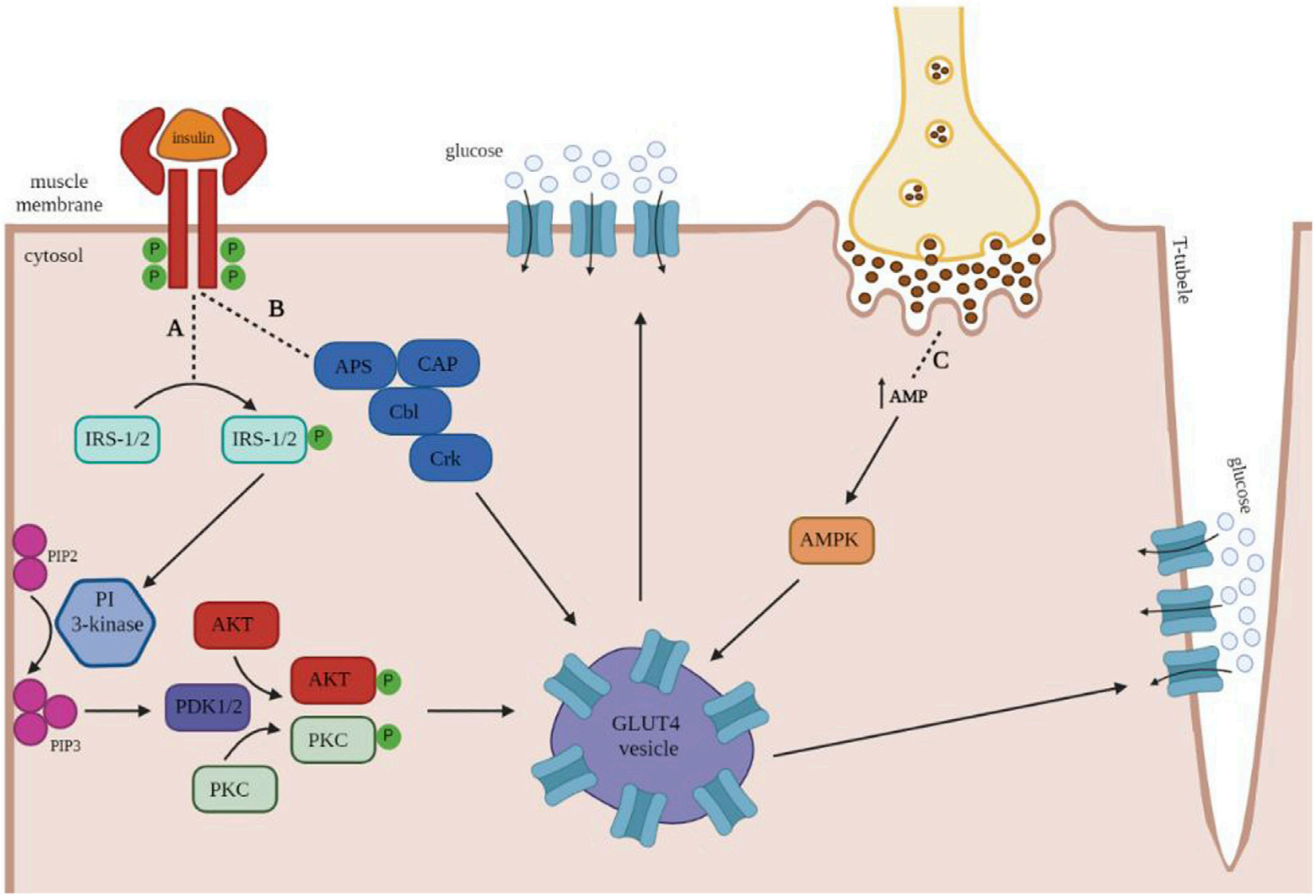
Insulin and muscle contraction mediated translocation of GLUT4 in human skeletal muscles. Once insulin binds to its receptor, it leads to the autophosphorylation of the transmembrane units, which in turn activates the downstream signaling proteins insulin receptor substrate 1 and 2 (IRS1/2) and adapter protein with Pleckstrin and Src homology domains (APS). **(A)** Phosphorylated IRS1/2 proteins serve as an activator of the phosphatidylinositol (PI)- 3-kinase, which converts PIP2 to PIP3 at the plasma membrane. Further, PIP3 activates the phosphoinositide-dependent protein kinases 1 and 2 (PDK1/2), which phosphorylate protein kinase B (AKT) and protein kinase C (PKC). Both kinases contribute to the trafficking of GLUT4-containing vesicles toward the plasma membrane. **(B)** Activation of the insulin receptor also increases phosphorylation of the proto-oncogene B-lineage lymphoma (Cbl), a process that requires the presence of the adaptor proteins APS and CAP. The Cbl-APS/CAP complex enhances the recruitment of CT10 regulator of kinase (Crk), which has been proposed to promote GLUT4 translocation to the plasma membrane. **(C)** In order to fulfill the increased energy demands of skeletal muscle during exercise, muscle contraction increases 5′ adenosine monophosphate (AMP) levels, leading to the activation of AMP kinase (AMPK), which translocates GLUT4-containing vesicles towards the plasma membrane and the T-tubules. Image generated with biorender.com.

Isotype GLUT4 is the most intensely studied GLUT isotype in humans and rodents since it is primarily responsible for insulin-dependent glucose uptake into tissues (Marette, 2003; Shao and Tian, 2015). In the absence of insulin, GLUT 4 is mainly stored intracellularly in vesicles. Both insulin and muscle contraction stimulate the translocation and insertion of GLUT4 into the cell membrane (Vargas et al., 2023). Figure 1 illustrates the insulin-mediated and muscle contraction-mediated GLUT4 translocation pathways.

Interestingly, the impact of insulin on membranal GLUT4 expression seems species-dependent since GLUT4 translocation in skeletal muscles increased from 80% to 400% after *in vivo* insulin stimulation in humans, whereas in horses, this test minimally increased GLUT4 translocation (15%) (Ryder et al., 2000; Duehlmeier et al., 2010; Waller et al., 2011). Furthermore, in a study by Katz et al. (1995), the role of GLUT4 in glucose homeostasis was assessed by disrupting the murine GLUT4 gene. The mice showed cardiac and adipose tissue metabolic abnormalities but did not develop a hyperglycemia phenotype after oral glucose feeding (Katz et al., 1995). These data suggest that other GLUTs, besides GLUT4, can be involved in regulating whole-body glucose homeostasis. As mentioned in Table 1, GLUT8, a dual-specific glucose and fructose transporter, has been shown to participate as another insulin-dependent GLUT isotype in humans (Aerni-Flessner et al., 2012). Protein GLUT8 is expressed in the striated muscles and the digital lamellae of horses (De Laat et al., 2015a). In addition, GLUT12 is another novel insulin-sensitive GLUT isotype, although its function may be more tissue-specific than other isotypes (Pujol-Giménez et al., 2013). In human skeletal muscle, it has been shown that insulin can also promote the translocation of GLUT12 from an intracellular location to the plasma membrane (Macheda et al., 2005; Wilson-O’Brien et al., 2008; Stuart et al., 2009). Furthermore, equine GLUT12 is expressed in insulin-sensitive tissues, with a similar tissue distribution pattern as GLUT4 (Lacombe, 2014).

In humans and rodents, there is evidence that exercise also changes GLUT8 and GLUT12 expression levels (Seki et al., 2006; Stuart et al., 2010; Narita et al., 2019). For instance, trained athletes that performed a single bout of exercise showed lower GLUT12 mRNA expression in their m. vastus lateralis (VL) when compared to sedentary individuals that performed the same exercise protocol (Seki et al., 2006). In mice, 3 days of training, after being supplemented with trehalose for 4 days, triggered an increase of GLUT8 protein levels in the brain and the liver (Narita et al., 2019). In a longitudinal follow-up study in untrained humans, 6 weeks of training increased GLUT12 protein levels by 104% compared to basal levels in the VL (Stuart et al., 2010). However, studies investigating the effect of exercise and/or training on glucose transporter expression are very scarce in horses. Most equine-related research has been focusing on impaired glucose uptake in the context of pathological conditions, such as equine metabolic syndrome (EMS), which can more or less be compared to Type II diabetes (T2DM) in humans and rodents (Walsh et al., 2009; Morgan et al., 2015; 2016; Durham et al., 2019). A core hallmark in the development of T2DM is insulin resistance (IR), which is also associated with obesity (Abdul-Ghani and Defronzo, 2010; Dumba et al., 2015). In horses, IR is the major catalyst of the development of EMS. Horses suffering from EMS develop a typical phenotype defined by obesity, hyperinsulinemia, hyperglycemia, and an increased risk for development of laminitis, a life-threatening disease in horses (Durham et al., 2019). Various exercise programs have been developed in an attempt to prevent the exacerbation of symptoms shown by horses suffering from EMS (Walsh et al., 2009; Morgan et al., 2015; 2016). Implementing physical activity in horses with EMS also showed to be beneficial for modulating insulin sensitivity (Morgan et al., 2016). However, the underlying effects of exercise on glucose transport dynamics, both in healthy horses and horses suffering from EMS remains unclear (Lacombe, 2014). Therefore, the aims of the current study were to quantify expression levels of three different GLUT isotypes (GLUT4, 8, and 12) in locomotion (m. vastus lateralis, VL) versus posture muscles (m. pectoralis, PM) in healthy untrained horses and to assess the effect of acute exercise and training on muscle GLUT4, GLUT8, and GLUT12 expression in healthy horses.

## Materials and methods

### Animals

A homogenous group of sixteen untrained Standardbred mares (aged 3–5 years) was enrolled. All horses were housed at the same training stable in individual boxes on straw bedding. Horses had *ad libitum* access to tap water and good quality hay (>1 kg hay/100 kg BW) and were fed an isocaloric diet twice a day (at 8 a.m. and 8 p.m.). Prior to the start of the training trial, an acclimation period of 2 weeks was allowed. Body weight was measured before (avg. 453 kg ± 31 kg) and after (avg. 449 kg ± 36 kg) the training trial. Likewise, body condition scoring was performed, which remained the same throughout the entire training trial (avg. 4.5 ± 0.5 on a scale of 1–9). Horses were regularly monitored by a veterinarian and vital signs were checked twice daily, including pulse, temperature, respiratory rate, capillary refill time, appetite, and stool production. There were no adverse events throughout the trial and the horses stayed healthy throughout the entire training trial.

### Training regime

**FIGURE 2:**
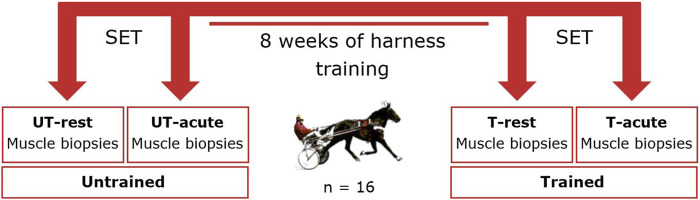
Overview training trial protocol and sample collection timepoints. SET: standardized exercise test; UT-rest: untrained at rest; UT-acute: untrained after execution of SET; T-rest; trained at rest; T-acute: trained after execution of SET (Standardized Exercise Test).

Figure 2 provides an overview of the training trial protocol. The training protocol was the same for each week, including 4 days of training and 3 days of rest. Briefly, the same experienced driver harness trained each horse for eight consecutive weeks on the same oval-shaped sand racetrack. Horses were equipped with a GPS tracker and heart rate monitor (Polar® Equine H7, Polar Electro Oy, Finland). Each training session started with a 10-min warming-up (±20 km/h), followed by either 30 min of aerobic training (3 days a week, ±25 km/h, mean HR ± 150 BPM) or 3 × 3 min interval training (day 4) at high speed (±35 km/h). The horses performed an incremental standardized exercise test (SET) in the afternoon, at the start and end of 8 weeks harness training. Briefly, horses were warmed-up for 10 min at 20 km/h followed by 5 incremental exercise bouts of 3 min duration (an increase of 4 km/h every exercise step) until a 40 km/h velocity was reached. Muscle biopsies were harvested from the PM and the VL before and after each SET, which allowed for comparing rest and acute samples in untrained (UT) and trained (T) conditions (UT-rest vs. UT-acute vs. T-rest vs. T-acute). The study was approved by the Animal Ethics Committee of Ghent University (EC 2016/40).

### Muscle biopsies

Muscle biopsies were harvested from the m. pectoralis (PM) and the m. vastus lateralis (VL). First, horses were sedated with detomidine (10 μg/kg BWT) (Detogesic 10 mg/mL, Vetcare) and butorphanol (20 μg/kg BWT) (Butomidor 10 mg/mL, Richter Pharma AG). The muscle area was clipped, shaved, and disinfected. For local anesthesia, an ointment (Emla 5%, Astra-Zeneca) was applied, and after 10 min, lidocaine (lidocaine HCl 10 mg/mL, B. Braun) was injected subcutaneously. A surgical blade number 11 was used to pierce a small incision, after which a needle (14G Bergström) was inserted into the muscle until a depth of 4 cm was reached. Samples were taken under suction pressure and immediately placed in Tissue-Tek® O.C.T compound (Sakura Finetek) and snap-frozen in liquid nitrogen-cooled isopentane (Sigma-Aldrich). Samples were stored at −80°C until further processing after finalization of 8 weeks training.

### Protein extraction and quantification

In total, 12 mg of frozen muscle was added to an Eppendorf tube containing 400 µL lysis buffer, Triton X-100 (Sigma-Aldrich, X100), and PierceTM protease inhibitor (Thermo Scientific, 88,666). Disruption and homogenization were done using a Polytron device (Kinematica Polytron™ PT1200E Handheld Homogenizer) for 3 × 10 secs. At 4°C, samples were rotated for 2 h and centrifuged for 30 min. The supernatant was collected, and protein quantification of muscle lysates was done using the BSA protein assay kit (Thermo Scientific, 23,225) according to the manufacturer’s instructions. Because of problems with the lysis buffer, protein extraction of the PM and the VL failed for four horses out of sixteen (protein 260/280 purity ratio >0.65).

### Western blot

Muscle lysates (15 μg/μL) were prepared in 2x Laemmli sample buffer (Sigma-Aldrich, S3401). Electrophoresis was performed using 12% SDS-PAGE gel in 1x Tris-glycine SDS running buffer (Bio-Rad, 1610732). Afterwards, proteins were transferred from the gel onto mini trans-blot filter papers (Bio-Rad, 1703932EDU) using transfer buffer (25 mM tris, 192 mM glycine, and 20% v/v methanol). Blocking of filter papers was done at room temperature using 10% milk (GLUT4, GLUT8, and GLUT12) or 5% bovine serum albumin (Glyceraldehyde-3-phosphate dehydrogenase/GAPDH) in Tris-buffered Saline with 0.1% Tween 20 (TBST; Sigma-Aldrich, T9039). Filter papers were incubated overnight at 4°C on a shaker, with primary polyclonal antibodies diluted in 10% milk TBST (GLUT4, GLUT8, and GLUT12) or 5% bovine serum albumin TBST (GAPDH). Afterwards, filter papers were washed 5 × 5 min with TBST, incubated for 45 min with goat anti-rabbit peroxidase secondary antibody diluted in 10% milk TBST, and again 5 × 5 min washed with TBST. Detection of secondary antibodies was done using SuperSignalTM West Atto ECL Substrate (Thermo Scientific, A38554) and ChemiDoc MP Imaging System (Bio-Rad). The quality of loaded western blot gels was checked and excluded for analysis if background noise was too high. The total protein expression of GLUT4, GLUT8, and GLUT12 was determined with Image Pro1 analyzer software (Media Cybernetics Inc., Rockville, United States). Data was normalized against the housekeeping protein GAPDH. Results are provided in arbitrary units (a.u.).

### Statistical analysis

Since normality (Shapiro-Wilk’s test) of the data could not be assumed (VL: all P_GLUT4_ ≥ 0.9386; all P_GLUT8_ ≥ 0.9477; all P_GLUT12_ ≥ 0.9473; PM: all P_GLUT4_ ≥ 0.8889; all P_GLUT8_ ≥ 0.8575; all P_GLUT12_ ≥ 0.9130), data were analyzed using nonparametric tests. Differences in basal GLUT4, GLUT8, and GLUT12 levels between the PM and VL were analyzed with the Wilcoxon matched-pairs signed rank test. The effect of acute exercise and/or training on total GLUT4, GLUT8, and GLUT12 protein expression in the PM and the VL was analyzed using the Friedman test with a *post hoc* Dunn’s test (selected multiple comparison: UT-rest vs. UT-acute; T-rest vs. T-acute; UT-rest vs. T-rest; UT-rest vs. T-acute; UT-acute vs. T-acute). Significance level was set at *p* < 0.05.

## Results

### Basal GLUT4, GLUT8, and GLUT12 expression in the PM versus the VL muscle

**FIGURE 3:**
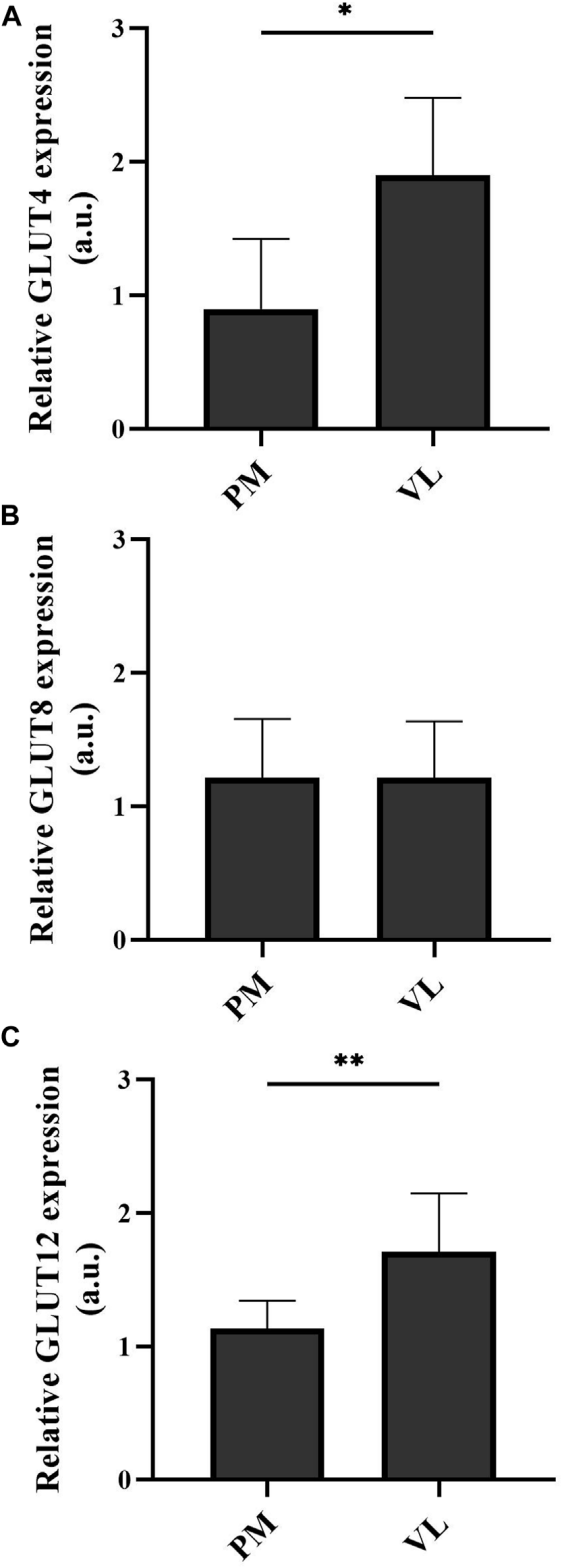
Basal GLUT4 **(A)**, GLUT8 **(B)**, and GLUT12 **(C)** protein expression in the m. pectoralis (PM) *versus* the m. vastus lateralis (VL). Graphs represent mean ± standard deviation of total protein expression in arbitrary units. *p < 0.05; **p < 0.01. a.u. arbitrary units.

Basal GLUT4 and GLUT12 protein expression was significantly higher in the VL compared to the PM (P_GLUT4_ = 0.0313 and P_GLUT12_ = 0.0020, Figures 3A, C, respectively). No significant difference was detected in basal GLUT8 protein expression between the two examined muscle groups (*p* > 0.9999) (Figure 3B).

### Total GLUT4, GLUT8, and GLUT12 expression dynamics in the PM and the VL muscle in response to training and/or acute exercise

#### Total GLUT4 protein expression in the PM and the VL in response to training and/or acute exercise

**FIGURE 4:**
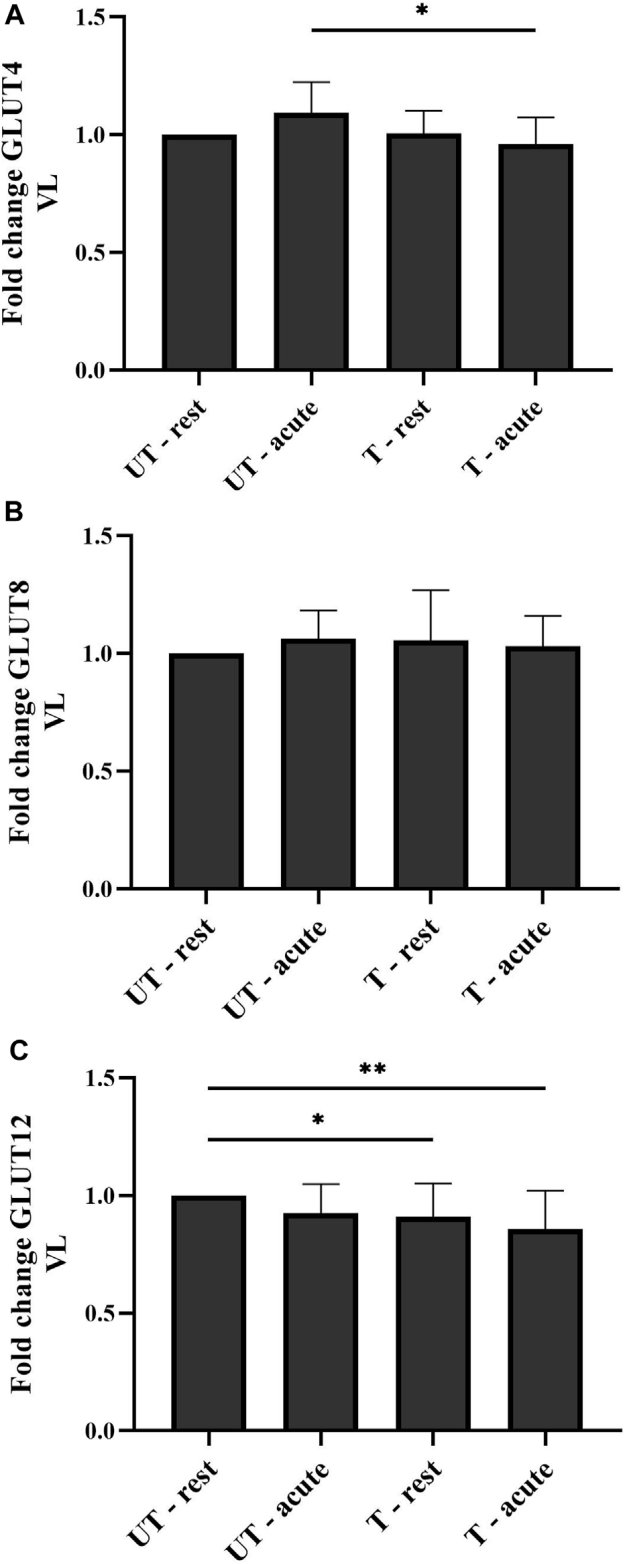
Total GLUT4 **(A)**, GLUT8 **(B)**, and GLUT12 **(C)** protein expression in the m. vastus lateralis (VL) in response to acute exercise and 8 weeks of harness training. Graphs represent mean ± standard deviation of total protein expression, relative to basal levels (UT-rest) in fold change. *p < 0.05; **p < 0.01. UT: untrained; T: trained.

Eight weeks of harness training had no effect on basal GLUT4 expression, neither in the VL (*p* > 0.9999), nor in the PM (*p* > 0.9999). However, acute exercise in trained condition significantly decreased GLUT 4 expression in the VL (*p* = 0.0148), but not in the PM (*p* > 0.9999) (Figure 4A). In both examined muscle groups, acute exercise in neither UT (P_PM_ > 0.9999 and P_VL_ = 0.6860) nor T (P_PM_ > 0.9999 and P_VL_ = 0.3464) conditions significantly changed total GLUT4 protein levels compared to UT-rest and T-rest conditions, respectively. No significant difference was detected in total GLUT4 protein expression in the VL (*p* = 0.6860) and the PM (*p* > 0.9999) when comparing UT-rest and T-acute conditions.

### Total GLUT8 protein expression in the PM and the VL in response to training and/or acute exercise

Neither 8 weeks of harness training nor acute exercise in UT and T conditions significantly changed total GLUT8 protein expression levels in both the VL (P_UT-rest_ > 0.9999; P_T-rest_ > 0.9999; P_UT-acute_ > 0.9999; P_T-acute_ > 0.9999) and the PM [(P_UT-rest_ > 0.9999; P_T-rest_ > 0.9999; P_UT-acute_ > 0.9999; P_T-acute_ > 0.9999) (Figure 4B).

### Total GLUT12 protein expression in the m. pectoralis and the m. vastus lateralis in response to training and/or acute exercise

Eight weeks of harness training significantly decreased total GLUT12 protein expression in rest biopsies, only visible in the VL (*p* = 0.0359) (Figure 4C), and not in the PM (*p* > 0.9999). This decrease in GLUT12 expression was even more prominent in the VL after acute exercise in trained condition (P_VL_ = 0.0025 and P_PM_ > 0.9999) (Figure 4C). Compared to UT-rest and T-rest conditions, acute exercise in UT and T conditions did not significantly change total GLUT12 protein expression in the VL (P_UT_ = 0.7736 and P_T_ > 0.9999) and the PM (P_UT_ > 0.9999 and P_T_ > 0.9999). In both muscle groups, no significant difference was detected in total GLUT12 protein expression when comparing the acute condition before and after training (P_VL_ = 0.1992 and P_PM_ > 0.9999).

## Discussion

In this study, skeletal muscle GLUT4, GLUT8, and GLUT12 protein expression levels were examined in healthy horses in response to acute exercise and 8 weeks of harness training. To the best of the authors’ knowledge, this is the first equine study that examined multiple GLUT isotype expression across muscle groups involved in either locomotion or posture. Study results showed that baseline GLUT4 and GLUT12 protein expression clearly differs between the examined muscle groups, in favor of a higher baseline expression in the locomotion muscle: the m. vastus lateralis. From all studied GLUT isotypes, GLUT12 showed the most pronounced differential changes in response to acute exercise and 8 weeks of harness training. Eight weeks of harness training significantly downregulated total GLUT12 protein expression in the m. vastus lateralis in rest conditions, which was even more pronounced after acute exercise in trained condition (Figure 4). The GLUT4 isotype showed a significant decrease in protein expression in the m. vastus lateralis after acute exercise in trained condition, although 8 weeks of training did not have any effect on GLUT4 protein expression in resting conditions (Figure 4). Throughout the entire training trial, the m. pectoralis showed no differential changes in either GLUT4, GLUT8, or GLUT12 protein expression, in either of the studied conditions (UT-rest, UT-acute, T-rest, T-acute). Finally, GLUT8 showed no differential changes in any of the studied conditions in any of the involved muscle groups.

Training studies involving a combination of locomotion versus posture muscles are scarce (White et al., 2017). Exercise studies mainly involve the m. gluteus medius (McCutcheon et al., 2002; Lacombe et al., 2003; Nout et al., 2003; Stewart-Hunt et al., 2006; Pratt et al., 2007). Due to the anatomic position of the m. pectoralis, this muscle group mainly functions to stabilize the proximal forelimbs of the horse (Payne et al., 2005b). This was also supported by a previous study, which showed that 8 weeks of harness training did not affect any of the studied muscle plasticity parameters in the m. pectoralis such as muscle fiber type composition, muscle fiber specific and mean fiber cross sectional area, mitochondrial density, and capillarization in Standardbred horses, the same breed that is involved in the current study (de Meeûs d’Argenteuil et al., 2021b). The lack of changes in GLUT4, GLUT8 and GLUT12 expression in the m. pectoralis of Standardbred horses in the current study, confirms the findings of the aforementioned study (de Meeûs d’Argenteuil et al., 2021b). The m. vastus lateralis, which is the primary part of the quadriceps femoris muscle, is involved in extending the stifle and knee in horses (Robert et al., 2002; Payne et al., 2005a; de Meeûs d’Argenteuil et al., 2021a). It is thus crucial to realize that skeletal muscle groups can cover different physiological roles and adapt differently in response to training (de Meeûs d’Argenteuil et al., 2021b). The current results also show variation in baseline characteristics between predominant posture versus locomotion muscle groups, since baseline GLUT4 and GLUT12 protein expression was significantly higher in the m. vastus lateralis compared to the m. pectoralis. This suggests the existence of essential differences in basal metabolic blueprint between posture versus locomotion muscle groups. It suggests that glucose metabolism is more modulated in locomotion muscles when compared to posture muscles, most probably because locomotory muscles need to have access to a much more comprehensive range of metabolic fuels, as they need to realize a much greater variety of exercise types in terms of both duration and intensity.

In the current study, GLUT expression was expected to increase both in response to acute exercise and training, if glucose metabolism were to play a pivotal role.

A first remarkable finding in the current study was the fact that 8 weeks of harness training did not affect total GLUT4 expression in the m. vastus lateralis, though a significant decrease was detected in total GLUT4 expression after acute exercise, only in trained condition (Figure 4). This is in contrast to human athletes and rodents in which it is well known that acute exercise and training increase GLUT4 expression on both mRNA and total protein levels in locomotory muscles (Hickner et al., 1997; Manolescu et al., 2007; Flores-Opazo et al., 2020; Vargas et al., 2023). However, in horses, neither acute exercise nor 8 weeks of harness training increased total GLUT4 protein expression in the m. vastus lateralis; on the contrary, a significant decrease was even observed after acute exercise in trained condition. These results align with other equine studies with that respect (Nout et al., 2003; Pratt et al., 2007; Manso Filho et al., 2017; Filho et al., 2020; Valberg et al., 2023). In a study by Nout et al. (2003), muscle biopsies from the m. semitendinosus of six horses were harvested before treadmill exercise and at multiple timepoints post-exercise. Neither in acute condition, nor in post-exercise rest condition significant changes in GLUT4 mRNA and total protein expression were detected (Nout et al., 2003). This was also confirmed in a study by Pratt et al. (2007), in which a single bout of acute treadmill exercise had no significant effect on total GLUT4 protein expression at the level of the m. gluteus medius in eight horses (Pratt et al., 2007). Similarly, in a more recent study, Manso Filho et al. (2017) reported that 8 weeks of training in a horse walker had no effect on GLUT4 protein expression in the m. gluteus medius of twelve horses (Manso Filho et al., 2017). The same research group performed a twelve-week longitudinal follow-up study on eight horses using the same horse walker, where not only there were no changes in GLUT4 protein expression in the m. gluteus medius, but also in other biochemical markers such as lactate and glucose (Filho et al., 2020). Likewise, Valberg et al. (2023) did not find an effect of acute exercise, nor during the 72 h glycogen repletion period, on GLUT4 gene expression in the m. gluteus medius of six horses after 3 months of training on a treadmill (Valberg et al., 2023). Interesting to notice is the fact that a few older studies indeed showed significant increases in skeletal muscle total GLUT4 protein levels as a response to exercise and/or training in horses (McCutcheon et al., 2002; Lacombe et al., 2003; Jose-Cunilleras et al., 2005; Stewart-Hunt et al., 2006). In a study performed by Lacombe et al. (2003), six horses underwent a short treadmill training protocol of 3 days, during which muscle biopsies of the m. gluteus medius were collected on a rest day pre-exercise and directly after the last treadmill exercise. Total GLUT4 protein expression was significantly increased in response to 3 days of treadmill exercise (Lacombe et al., 2003). This result was also reported in a study by Stewart-Hunt et al. (2006), in which nine horses were trained on a treadmill for seven consecutive days (Stewart-Hunt et al., 2006). Furthermore, when horses were trained on a treadmill for 6 weeks, total GLUT4 protein expression in the m. gluteus medius was significantly increased, in both rest and acute phase (McCutcheon et al., 2002). Similarly, Jose-Cunilleras et al. (2005) reported an increase in GLUT4 mRNA transcripts in the m. gluteus medius of seven horses 4 h post exercise following 3 days of training (Jose-Cunilleras et al., 2005). More recently, in a study with Alaskan sled dogs, an increase in GLUT4 protein expression was observed in the m. biceps femoris after 7 months of athletic conditioning (Barrett and Scott Davis, 2023). Of interest is the fact that studies that report an increase in GLUT 4 expression in response to training, all start off with horses/dogs that were not involved in any type of daily exercise (for example horses in these studies were either box rested or paddock rested) prior to start of the study and encompass rather short periods of training, ranging from 3 days to 6 weeks (McCutcheon et al., 2002; Lacombe et al., 2003; Jose-Cunilleras et al., 2005; Stewart-Hunt et al., 2006). More research is needed with that respect. It seems that demanding performance of intense exercise in such conditions does indeed launch important involvement of glucose metabolism and most probably associated lactic acid production.

In view of the fact that species differences exist with respect to GLUT expression and that also other insulin driven GLUTs exist, additionally GLUT12 and GLUT8 were involved in the current study. Clearly, the GLUT12 isotype was the most differentially influenced throughout the entire study, when compared to the other studied GLUT isotypes. Total GLUT12 protein levels decreased significantly in response to 8 weeks of harness training, and this decrease was even more pronounced after performing acute exercise in trained condition (Figure 4). It is also important to notice that GLUT4 expression followed the same trend, however, only after acute exercise in trained condition. Indeed, training *per se*, did not affect total GLUT4 expression in the current study. Interestingly, up until now, GLUT12 has been seen as the evolutionary GLUT4 ancestor, although there is increasing evidence to refute such hypotheses (Pujol-Giménez et al., 2015). Finally, GLUT8 expression did not show any changes in any of the studied conditions.

To the best of the authors’ knowledge, this is the first study to examine the effect of acute exercise and 8 weeks of harness training on total GLUT4, 8 and GLUT12 protein expression in the m. vastus lateralis and the m. pectoralis of horses. The results related to both GLUT8 and GLUT12 align with results found in the m. vastus lateralis of humans and the m. biceps femoris of dogs (Seki et al., 2006; Barrett and Scott Davis, 2023), suggesting that GLUT8 does not play an essential role in skeletal muscle metabolism during exercise and training. A study by Seki et al. (2006) examined the differences in GLUT8 and GLUT12 mRNA expression in the m. vastus lateralis from fifteen untrained and sixteen trained human subjects at rest. No significant difference in GLUT8 mRNA expression between the two examined groups was detected, while GLUT12 mRNA expression was significantly lower in the trained group compared to the untrained group (Seki et al., 2006). The same researchers observed similar results in eleven men and five women, who performed a running, cycling, and swimming training program versus a sedentary group of eleven men and four women. After the training protocol was completed, muscle biopsies were harvested from the m. vastus lateralis of all volunteers and GLUT8 and GLUT12 mRNA expression was assessed. Again, significantly lowered GLUT12 mRNA levels were detected in the trained group compared to the sedentary group, while no significant difference was detected in GLUT8 mRNA levels (Seki, 2004). However, on protein level, total GLUT12 expression seems to increase in response to exercise in humans. In a study by Stuart et al. (2010) six untrained volunteers completed 6 weeks of cycle training, in which the power output was increased over the training period. Muscle biopsies of the m. vastus lateralis were taken pre-and post-training at rest moments to determine total GLUT12 protein expression, which increased 104% in response to cycle training (Stuart et al., 2010). Furthermore, in skeletal muscles of twelve Alaskan sled dogs, total GLUT12 protein expression was increased after 7 months of progressive endurance conditioning, while no significant change was measued in total GLUT8 protein expression (Barrett and Scott Davis, 2023). In horses, only one study is available that determined total GLUT8 protein expression in skeletal muscles in response to AMPK activation. Muscle biopsies of the m. semitendinosus of five horses were taken before and after intravenous administration of 5-aminoimidazole-4-carboxamide-1-D ribofuranoside (AICAR). AICAR is defined as an AMPK activator, which significantly increased total GLUT8 protein expression in the m. semitendinosus (de Laat et al., 2015b). This result could suggest that GLUT8 plays an important role in equine skeletal muscle glucose transport. However, as seen in human-related studies and the current study results, neither acute exercise nor 8 weeks of harness training changed total GLUT8 protein expression levels in the m. vastus lateralis and the m. pectoralis, while GLUT12 expression was significantly downregulated in the m. vastus lateralis in response to 8 weeks of harness training and acute exercise.

When focusing in on GLUT8 and GLUT12 at the ultrastructural level, with respect to tissue localization and their capacity to function as gateway for different fuels, a lot has been learnt the past decades. In humans, GLUT8 is predominantly expressed in brain, liver, and testes, while GLUT12 is predominantly expressed in adipose tissue, cardiac muscle and placenta (Aerni-Flessner et al., 2012; Pujol-Giménez et al., 2013). The GLUT12 isotype has also been seen present in the apical region of distal tubules and collecting ducts in the kidney and epithelial cells of the jejunum, chromaffin cells, the anterior pituitary lobe and thyroid and pyloric glands in mice. This tissue distribution suggests a unique function of GLUT12, besides that of an insulin-dependent glucose transporter (Matsuo et al., 2020).

In recent years, more research has been done to investigate the function of GLUT8 and GLUT12 in various pathophysiological conditions, such as the role of GLUT8 in nonalcoholic fatty liver disease (NAFLD) and GLUT12 in tumor growth and metastasis. Additionally, GLUT12 has been shown to transport a wide diversity of hexoses aside from glucose, such as galactose, fructose, α-methyl-D-glucopyranoside and 2-deoxy-D-glucose (Pujol-Giménez et al., 2015). This transporter can also work as a Na^+^ or H^+^/glucose symporter and it shows electrogenic properties (Wilson-O’Brien et al., 2010; Pujol-Giménez et al., 2013; 2015). A study by DeBosch et al. (2014) measured reduced fructose uptake in hepatocytes of GLUT8 knock-out mice, which protected the mice from fructose-induced intrahepatic lipid accumulation (steatosis), a key mechanism in the pathogenesis of NAFLD (DeBosch et al., 2014).

In a human study, a triple negative breast cancer (TNBC) cell line was cultured and GLUT12 was knocked down. Cell proliferation, migration, and invasion was reduced in GLUT12 knock-down TNBC cells compared to a control TNBC cell line, while re-expression of GLUT12 avoided these effects. In addition, suppression of GLUT12 in TNBC cells led to decreased glucose uptake and increased production of pyruvate, lactate, and ATP, while mitochondrial respiration was increased. Re-activation of GLUT12 reversed these effects. Together, these results suggest that GLUT12 promotes TNBC tumor growth and metastasis through the Warburg effect (Shi et al., 2020).

From a physiological viewpoint, important changes in GLUT expression should be expected if the glucose influx plays a pivotal role in fueling or connecting metabolic pathways that are upregulated in certain conditions, such as acute exercise versus training. The absence of upregulation in any of the studied GLUT isotypes and even downregulation of GLUT12 and, to a lesser extent, GLUT4 brings the critical role of glucose to question. Especially since in humans and rodents, increases in GLUT expression are linked to the role of glucose as key fuel in the working muscle cells (Scheepers et al., 2004; Evans et al., 2019). The current results suggest that, in horses, skeletal muscles probably use energy sources other than glucose to produce energy when performing acute exercise in trained condition. These results also explain why persistent low glycogen levels were measured in the muscles of trained horses (Essen-Gustavsson et al., 1989) and why it takes horses 2-3 times more time to replenish depleted muscular glycogen stores compared to other mammals (Reed et al., 1989; Lacombe et al., 2003; 2004). The present findings also confirm why dietary glycogen loading of the muscles is not beneficial in horses for achieving increased performance capacity, as opposed to humans (Iwayama et al., 2023). Most probably equine muscle cells switch to alternative energy pathways, other than glucose-pivotal ones when performing exercise and this is even more pronounced in trained condition. With that respect, the animal kingdom harbors many alternatives under certain circumstances, for example, disease or growth, some of which have already been unraveled, others are still subject of research. In horses, diabetes type I has not been reported, likewise, ketogenesis is very rare in horses (Naylor et al., 1980). Hence, it seems like the TCA cycle in horses can be fed by other fuels then glucose, most probably sideways, beyond the first Acetyl-CoA step.

In humans, there is a fifty-fifty distribution of slow and fast twitch fibers, whereas in horses, type IIA is the predominant fiber type (Zierath and Hawley, 2004; Rivero and Hill, 2016). Horses thrive on type IIA fibers, it’s the most important building block of their muscular compartment. Those are fast oxidative glycolytic fibers, which entails that they can produce ATP at high speed, however, in mainly aerobic conditions. It is hypothesized that this is achieved by increased mitochondrial tricarboxylic acid (TCA) cycle activity (Schiaffino and Reggiani, 2011; Arnold and Finley, 2023). Indeed, in the last step of glycolysis, pyruvate is formed, which serves, in the presence of oxygen, as a precursor for Acetyl-CoA, the starting substrate for the TCA cycle (Arnold and Finley, 2023). The results of the current study suggest that other substrates, besides glucose, can feed sideways into the TCA cycle or at least feed the type IIA fibers, which is the predominant fiber type in horses (Schiaffino and Reggiani, 2011; Arnold and Finley, 2023). In a previous study, applying the combination of muscle fiber typing with untargeted metabolomics in Friesian horses that were subjected to dry treadmill training during 8 consecutive weeks, the m. vastus lateralis was shown to expand its aerobic fast twitch profile, with decreased muscle diameter, decreased type I fibers and an upregulation of glycolytic and pentose phosphate pathway (PPP) activity, and increased branched-chain (BCAA) and aromatic amino acid (AAA) metabolism. The fact that only modest glycogen metabolism pathway changes were seen in that study brings to question whether CHOs are pivotal energy sources for horses being trained. Results show that BCAAs, AAAs, acylcarnitines and microbiome-derived xenobiotics need further study in horses. Several of these feed into the TCA cycle at steps further downstream from Acetyl-CoA and most likely are oxidized in type IIA fibers, the predominant fiber type of the horse. These study results again underline the importance of reviewing existing paradigms on equine bioenergetics.

One limitation of the current study is that total protein content was isolated, allowing for quantification of total GLUT4, GLUT8, and GLUT12 protein expression but not allowing for quantifying GLUTs available at the plasma membrane for glucose transport. However, other studies performed in humans, rodents, and horses also measured total GLUT4, GLUT8, or GLUT12 protein expression and confirmed that this approach is adequate to quantify changes in GLUT expression in response to exercise and training (McCutcheon et al., 2002; Pratt et al., 2007; Narita et al., 2019).

To conclude: from a physiological point of view, important changes in GLUT expression would be expected if the glucose influx plays a pivotal role in fueling metabolic pathways that are upregulated in certain conditions, such as acute exercise versus training. Locomotor muscles show significantly higher expression in GLUT4 and GLUT12 expression when compared to posture predominant muscles, confirming the fact that locomotion muscles can fall back on a wider array of metabolic routes in order to fulfill their complex locomotor function as compared to posture muscles. Within locomotor muscles, the GLUT12 isotype shows the most prominent differential changes: there is a significant decrease in expression in response to 8 weeks of harness training and this is even more pronounced when acute exercise is performed in trained condition. The GLUT4 isotype follows the same trend, where a significant downregulation is seen in response to acute exercise in trained condition, however, no effect of 8 weeks of training *per se* could be seen. The important changes seen in GLUT12 expression downregulation, both in response to training and acute exercise in the horse, the downregulation of GLUT4 expression after acute exercise in trained condition and the lack of differential shifts in GLUT8 expression in any of the studied conditions, questions the importance of glucose/glycogen as substrate to fuel training and exercise in horses. These findings encourage to further explore alternative fuels for their involvement in equine muscular energetics. Such knowledge will reveal groundbreaking views on alternative energy pathways, applicable to other animal species in both heathy and pathological conditions.

## Data Availability

The datasets generated for this study are available on request to the corresponding author.
